# Arthroscopic All-Inside Repair of Tear of the Anterior Horn of Discoid Lateral Meniscus

**DOI:** 10.1055/s-0039-1688951

**Published:** 2019-05-14

**Authors:** Moganadass Muniandy, Sivalingam Rajagopal, Siti Hawa Tahir

**Affiliations:** 1 Department of Orthopaedic, Hospital Kuala Lumpur, Malaysia

**Keywords:** anterior horn lateral meniscus, all-inside repair, outside-in repair, discoid meniscus

## Abstract

The anterior horn of lateral meniscus tear is usually repaired using outside-in technique. Although easy to perform, it was associated with several complications which may alter the outcome of the surgery. Here, we present a case of an adolescent girl presented with incomplete discoid lateral meniscus accompanied by tear of anterior horn. The tear was repaired using all-inside technique without any implants. We described the surgical technique and discussed the rationale.

The commonly used technique to repair anterior horn of lateral meniscus is the outside-in technique. Although safe and easy to perform, it is not without its complications and disadvantages. Here, we present a patient with tear of discoid lateral meniscus involving anterior horn, repaired using all-inside technique.

## Case Report

**Fig. 1:**
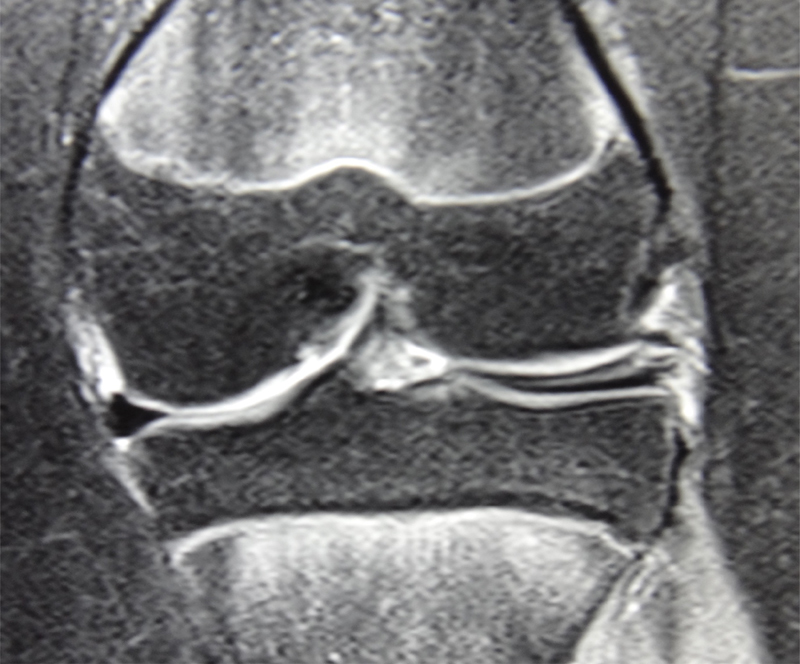
Coronal slice of MRI showing discoid lateral meniscus with complex horizontal tear. MRI, magnetic resonance imaging.

**Fig. 2:**
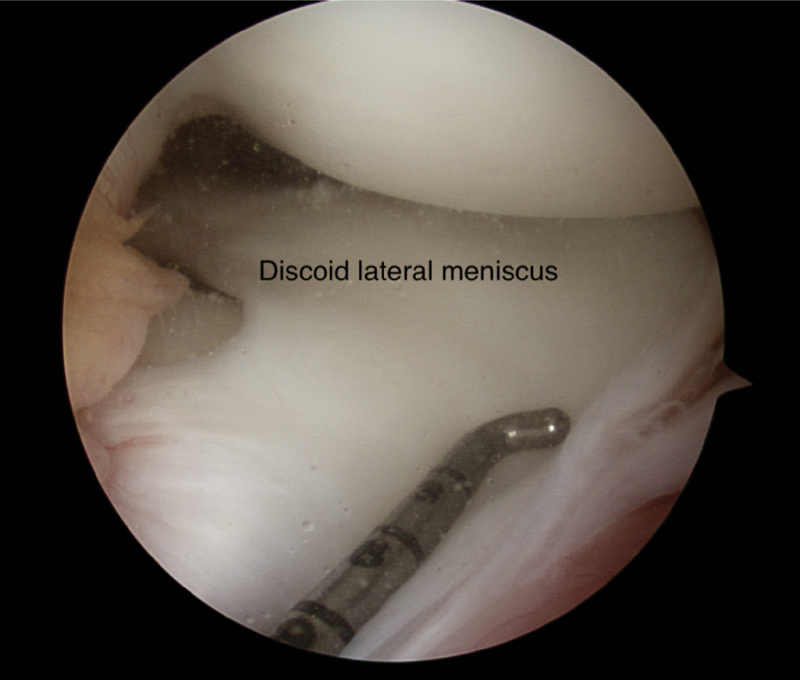
Incomplete discoid lateral meniscus with vertical tear involving anterior horn.

A 14-year-old girl, presented to our clinic with left knee pain, especially on full extension of the knee, for duration of 6 months. There was no trauma involved. Examination showed lateral joint line tenderness without any ligamentous laxity. Plain radiographs of the knee were normal. Further imaging with magnetic resonance imaging (MRI) scan revealed discoid lateral meniscus with a horizontal tear ( Fig. 1 ). Diagnostic scope was done and we found an incomplete discoid lateral meniscus with complex vertical tear involving anterior horn with horizontal extension into the midbody ( Fig. 2 ). Arthroscopic all-inside repair was done without any implant. Postoperatively, the patient's knee was protected with a brace. Postsurgery, 6 weeks, the brace was discontinued and the patient started full weight bearing without pain. At 6 months postsurgery, the patient was pain free and without any mechanical symptoms.

### Surgical Technique

**Fig. 3:**
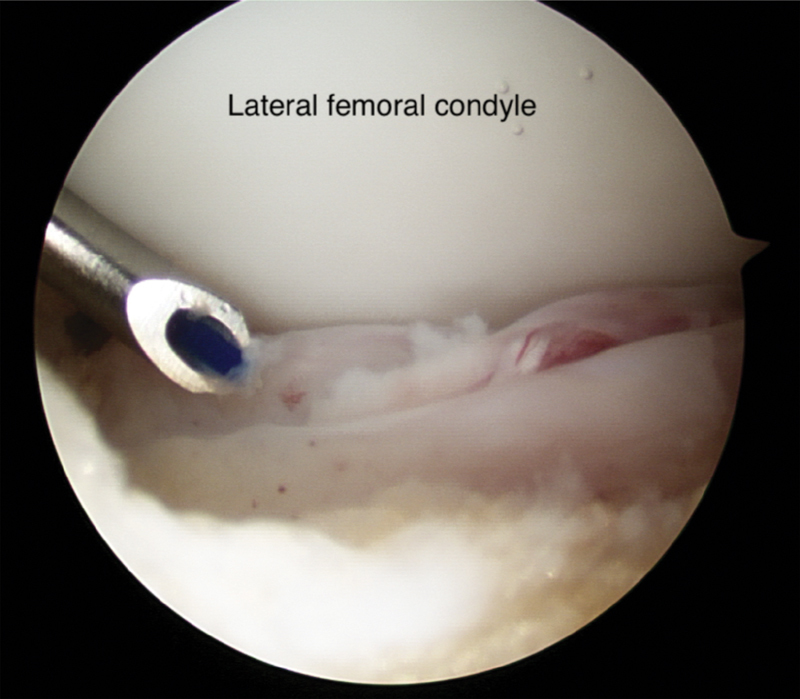
A suture passer loaded with monofilament absorbable suture.

**Fig. 4:**
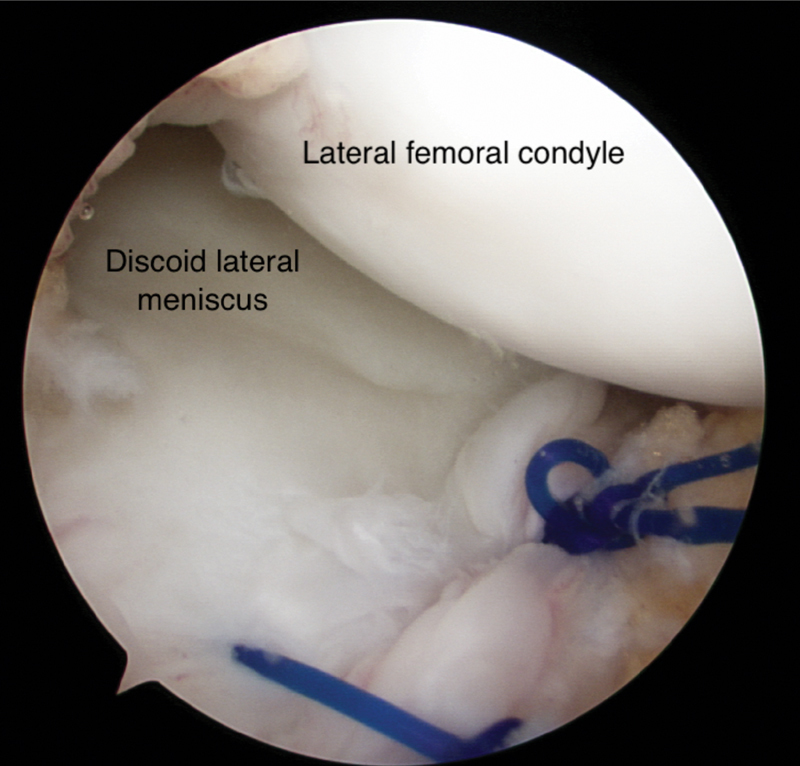
Completed repair of the tear.

The position was supine, with the leg hung freely at the end of the table. A tourniquet was used to secure hemostasis. A standard anterolateral portal was used for diagnostic arthroscopy. The finding was an incomplete discoid lateral meniscus with peripheral vertical tear of the anterior horn and partial horizontal tear involving the mid body but not breaching the inner peripheries ( Fig. 2 ). A standard anteromedial portal was made and the tear was further assessed using a probe. The tear site was prepared for repair. An additional far medial portal was made to allow arthroscopic instrumentation. A suture passer loaded with synthetic monofilament absorbable suture, introduced through far medial portal, while viewing through anteromedial portal ( Fig. 3 ). The torn central fragment and the peripheral rim of the meniscus were penetrated and the tip of suture was pulled through using an arthroscopic grasper through anterolateral portal. The suture passer was reversed out of the far medial portal, leaving the suture inside. Then, a suture retriever was introduced through anterolateral portal and used to retrieve the suture tip bringing both suture-ends out through one portal. Both the suture ends were tied using a sliding knot technique. This whole process was repeated for a second suture repair ( Fig. 4 ). The stability of the repair was assessed using a probe. Initially, we planned to saucerize the discoid meniscus together with meniscus repair. However, intraoperatively we decided not to saucerize the meniscus in view of the tear configuration. Due to the complex nature of the tear pattern (peripheral vertical tear with extensive horizontal tear through the meniscus body), saucerization would have made the articular surface of the meniscus a loose fragment or created a flap tear, which is more difficult to repair.

## Discussion

The anterior horn of lateral meniscus tear is recommended to be repaired using outside-in or inside-out repair technique. Rodeo 1 recommended outside-in technique to repair tears involving anterior horn of meniscus. Despite being easy to access the tear site using outside-in technique, it has few disadvantages. The need for additional skin incision to make a subcutaneous knot, knot irritation causing pain, superficial infection due to local acidity caused by suture degradation, and misdirected needle in the joint causing cartilage injury were few of the complications reported in the literature. Although there was no information in the literature regarding the incidence of these complications, these are recognized postoperative adverse events that need to be dealt with.

The lateral meniscus is more mobile compared with medial meniscus due to its fewer capsular attachments. 2 During flexion, lateral meniscus moves posteriorly. 3 In repairing anterior horn of lateral meniscus using outside-in method, if the knot tying is over tightened extra-articularly, the excursion of lateral meniscus would be limited. This changes normal anatomy and biomechanics of knee and may cause pain due to entrapment of joint capsule anteriorly.

These disadvantages can be overcome if the anterior horn tears were to be repaired using all-inside technique. We repaired the tear of the anterior horn of lateral meniscus in the same manner of repairing labral tear in the shoulder. The instruments used, to repair the tear were the shoulder arthroscopic surgery instruments.

Kim et al 4 described a similar technique of repairing tears of anterior horn of lateral meniscus. Using three portals, lateral patellofemoral axillary portal as the viewing portal, standard anterolateral, and far medial portal, the tear was approached through far medial portal with a 90 degrees angled hook loaded with no 2 Polydioxanone suture (PDS). The suture was retrieved through anterolateral portal and exchanged with Ethibond suture (Ethicon, Somerville, NJ). Then the sutures were tied using sliding knot. The technique was used in five patients and all of them improved significantly from their preoperative status.

Choi 5 described almost similar technique using only two portals. The author made anteromedial portal first as the viewing portal. It was made just anterior to medial femoral condyle, 3 cm medial to patella tendon, and 1.5 cm above the joint. Then the anterolateral portal made under arthroscopic visualization. The anterolateral portal was used to introduce the suture hook, retrieve the suture, and tie a sliding knot. The author used no.0 PDS suture for the repair.

Cho 6 described a repair technique using only two portals and 18-gauge spinal needle. After creating the high anteromedial portal for viewing and standard anterolateral portal for instrumentation, 18-gauge spinal needle used to penetrate the capsular portion, crosses the tear, and exits the surface of inner fragment of meniscus. Suture material is advanced and pulled into the joint using suture grasper. Then, the needle withdrawn to the level of subcutaneous and reinserted through the capsular side of the tear. The folded suture then pulled into joint. The sutures are tied to form a vertical mattress stitch.

While Kim et al used unconventional lateral patellofemoral axillary portal as viewing portal, and Cho and Choi used only two portals in their surgeries, we used far medial portal as accessory working portal in addition to the anteromedial portal as viewing portal and anterolateral portal as working portal. This far medial portal can be easily created using a needle as guide. Besides, using three portals compared with two portals would make suture management and knot tying easier. We used this technique as it was easy to perform and can avoid all the complications of outside-in technique. It provides an alternative method to repair anterior horn tears.

We planned to use this technique in our future encounters with meniscal anterior horn tears as we believe that this technique is safe, easy to perform, and results in favorable outcome. Our future project would be a case series showing the reproducibility and the functional outcome of this technique.

## Conclusion

All-inside repair technique provides an alternative and effective way to repair meniscus tear involving anterior horn. In our technique, using conventional anterolateral and anteromedial portal with additional far medial portal, we could perform a secure repair of discoid lateral meniscus tear involving anterior horn.

## References

[1] Rodeo S A Arthroscopic meniscal repair with use of the outside-in technique Instr Course Lect 2000 49 195 206 10829175

[2] Thompson W O Thaete F L Fu F H Dye S F Tibial meniscal dynamics using three-dimensional reconstruction of magnetic resonance images Am J Sports Med 1991 19 03 210 215, discussion 215–216 1867329 10.1177/036354659101900302

[3] Safran M R Soto G Meniscus: diagnosis and decision making surgical techniques Philadelphia, PA WB Saunders 2004 497 498

[4] Kim S J Jung K A Kim J M Kwun J D Baek S H Han J N Arthroscopic all-inside repair of tears of the anterior horn of the lateral meniscus Arthroscopy 2005 21 11 1399 1399 16325096 10.1016/j.arthro.2005.08.027

[5] Choi N H Meniscal repair for anterior horn tear of the lateral meniscus Arthroscopy 2006 22 10 11320 113200 10.1016/j.arthro.2006.01.020 17027413

[6] Cho J H Arthroscopic all-inside repair of anterior horn tears of the lateral meniscus using a spinal needle Knee Surg Sports Traumatol Arthrosc 2008 16 07 683 686 18204832 10.1007/s00167-007-0483-9

